# Evaluation of Coriolis Micro Air Sampling to Detect Volatile and Semi-Volatile Organic Compounds

**DOI:** 10.3390/molecules27196462

**Published:** 2022-09-30

**Authors:** Audrey Courtier, Benoit Roig, Stephane Cariou, Axelle Cadiere, Sandrine Bayle

**Affiliations:** 1 UPR Chrome, University of Nimes, Rue du Dr G. Salan, CEDEX 1, 30021 Nimes, France; 2 Laboratoire des Sciences des Risques (LSR), IMT Alès, 6 Av. de Clavières, 30100 Alès, France

**Keywords:** atmospheric pollutants, Coriolis micro, Nalophan^®^ bags, environmental detection

## Abstract

There are several analytical procedures available for the monitoring of volatile organic compounds (VOCs) in the air, which differ mainly on sampling procedures. The Coriolis micro air sampler is a tool normally designed for biological air sampling. In this paper, the Coriolis micro bio collector is used to evaluate its ability to sample organic contaminants sampling and detecting them when combined GC-MS. We also compare the use of the Coriolis micro with a standardized sampling method, which is the use of a lung box with a Nalophan^®^ bag. The results show that the Coriolis micro sampling method is suitable for the sampling of organic contaminants. Indeed, the Coriolis micro allows to sample and detect mainly semi-volatile molecules, while the lung box/Nalophan^®^ bags allow to sample more volatile molecules (highly volatile and volatile). These results were confirmed in the controlled air lab with a slight difference with the field. The simultaneous use of the both techniques allow to sample and detect a larger number of molecules with specific physicochemical properties to each sampling technique. In conclusion, the Coriolis micro can sample and detect volatile organic compounds present in air. We have shown that the development of alternative sampling methods and the use of non-target analysis are essential for a more comprehensive risk assessment. Moreover, the use of the Coriolis micro allows the detection of emergent molecules around the Thau lagoon.

## 1. Introduction

The increase of industrialization and urbanization leads to growing pollution in different compartments such as air, water, and soil. These contaminations can induce risks for human health and the environment. The World Health Organization (WHO) alerts that 13.7 million deaths per year are related to the environment and that 4.2 million of these are the result of exposure to outdoor air pollution [1]. Outdoor air pollution has been associated with many diseases such as childhood asthma [2], kidney diseases [3], lung cancer [4], and cardiopulmonary disease [5].

The most common outdoor and indoor air pollutants are volatile organic compounds (VOCs) which are defined as any compound of carbon in atmospheric photochemical reactions, according to the United States Environmental Protection Agency (US-EPA) [6]. VOCs can be natural and anthropogenic sources and some of them are known to be of health concern [7,8,9,10,11]. Outdoor air pollutants are a heterogenous class of molecules that include particles such as bioaerosols, semi volatile compounds (SVOCs), volatile (VOCs), and very volatile compounds (VVOCs). Categories of chemical pollutants are defined by specific properties. For example, a VOC is defined by a boiling point (BP) ranging from 50–100 °C to 240–260 °C, while a SVOC has a BP ranging from 240–260 °C to 380–400 °C according to WHO [12]. The WHO intentionally does not clearly define between VVOCs and VOCs, while the US-EPA defined VVOCs as compounds having a BP lower than 50–100 °C [13]. Particles are quantified via airborne particulate matter (PM) and may represent a danger to human health. PM consists of a heterogenous mixture of airborne solid and liquid particles that varies continuously in size and chemical composition. The chemical constituents of PM are highly heterogenous and include nitrate, sulfates, elemental and organic carbon, organic compounds, biological compounds, and metals [14].

The greatest challenge in humans and/or the environment assessing risk is to sample as many airborne pollutants as possible that may pose a risk. Several analytical methods for the determination of VOCs in the air exist, which differ mainly by the sampling procedures [15]. The sampling procedures consist of passive or diffusive sampling, active sampling, the use of canisters and bags, and online sampling [16]. Passive sampling is based on the molecular diffusion of molecules through a surface and does not detect the pollution peak. It allows the sampling of most VOCs but can also sample SVOCs and particles [17,18]. In some studies, passive sampling has been used for VVOCs but has shown an underestimation of their concentration [19]. Active sampling is preferable for sampling VVOCs [20]. Active sampling consists of passing a constant flow of air through a tube filled with sorbent to catch VOCs. Sorbents can be of different types (activated carbon, polymers, silica, etc.) with one or several types of sorbents [21]. The main difficulty of this type of sampling is to maintain the highly volatile molecules trapped on the sorbents and the inherent artifact, depending on the sorbents used [21]. A canister consists of an inert stainless steel spherical container of several liters (1 to 15 L). After sampling, an aliquot of gas is transferred to a pre-concentrator trap of thermal desorber. It is also possible to collect air in bags and different types are available, such as Tedlar^®^, Kynar^®^ or Nalophan^®^ bags. Canisters or bags are a useful alternative to sorbent tubes, particularly for ultra-volatile compounds [16], and they can also be used for sampling particles [22]. The last method, that is the one currently used, is online sampling. The air samples are drawn manifold directly into the sorbent focusing trap of the thermal desorber. This technique requires a complete analytical system at each sampling point, but reduces the errors due to possible reactions occurring during storage of the adsorbent tubes before analysis [16].

Each sampling method has its advantages and disadvantages, as discussed above. The challenge lies especially in the sampling of SVOCs and VVOCs, i.e., managing to sample a large number of molecules with different properties. After sampling, two types of analysis can be performed to identify contamination: targeted and/or non-targeted analyses. Targeted analysis is generally used in order to find specific pollutants, which are mostly regulated substances [23,24]. Conversely, non-targeted analyses allow the discovery of new contaminants of emerging concern without “a priori”. Currently, environmental health studies, particularly for risk assessment, emphasize non-targeted approaches. These techniques allow the detection of many molecules [25,26,27]. Consequently, the results provided by these techniques highlighted new molecules whose health impact was previously unsuspected [28]. The right combination of sampling and analytical method allows a more accurate risk analysis.

In order to detect a maximum of compounds around the study site, a new methodology, including an original sampling/analysis combination, was developed. For the sampling method, the Coriolis micro (Bertin instruments, France), normally used to concentrate microorganisms in the air, was assessed. Indeed, this device has the advantage of concentrating air particles in water at a high flow rate, reducing the sampling time. Moreover, this technique allows the recovery of hydrophilic molecules or SVOCs. To extract and concentrate compounds from the water, a SBSE/HSSE extraction was realized before performing a non-targeted analysis by GC/MS (Agilent 7890B gas chromatograph/Agilent 5977A mass spectrometer). These analyses are compared to a standardized sampling method (NF X 43-104 from 1995 recently adopted by the EN 13725:2022 standard)which is a lung box containing Nalophan^®^ bags, followed by desorption on multi-bed sorbent tubes, and a non-targeted GC-MS analysis. Comparisons were performed in an operational context, either in the field or under controlled conditions.

## 2. Results

### 2.1. Comparison of the Two Sampling Techniques

**Figure 1:**
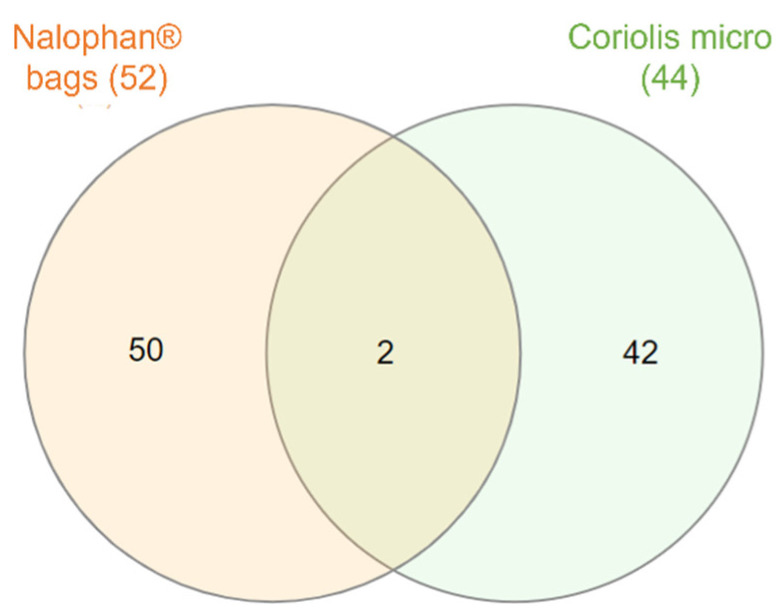
Distribution of molecules found using both techniques of air sampling. Venn diagram illustrating the degree of overlap of molecules found with Coriolis micro and Nalophan^®^ bags method. The central section in orange represents the compounds found in common between the two methods. Specific compounds found with Nalophan^®^ bags are in pink, whereas those found with Coriolis micro are in blue.

The air around the Thau lagoon was collected for 40 min using the lung box/Nalophan^®^ bag and the Coriolis micro techniques. The volumes of air collected were 40 L and 12,000 L, respectively. The air volume was higher with the use of the Coriolis. Fifty-two molecules were found with the Nalophan^®^ bags, while forty-four were found with Coriolis micro. Two molecules, nonanal and 2-ethyl-1-hexanol, were found in common (Figure 1). The volume of air sampled does not impact the number of molecules obtained but rather the nature of the molecules. Furthermore, it seems that the combination of both techniques allows to obtain a greater number of molecules. To understand which types of molecules were sampled according to the technique used, the physico-chemical properties of the molecules recovered with both techniques were sought (Supplementary Materials: Tables S1 and S2).

### 2.2. Compounds Properties

To assess the types of molecules retained by each of the two techniques, different physico-chemical parameters were investigated: molecular weight, the behavior in water compartment with solubility at 25 °C and the octanol/water partition coefficient, the behavior in air compartment with the octanol/air partition coefficient, Henry’s constant, boiling point, and vapor pressure.

#### 2.2.1. Molecular Weight

**Figure 2:**
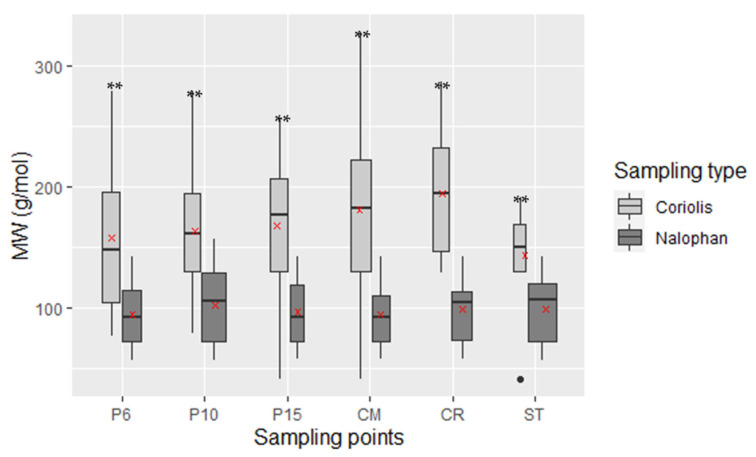
Comparison of molecular weights according to the sampler type used. The red crosses represent the mean values. MW are significantly different between two techniques (** mean *p* value < 0.01) values follow normal distribution except ST values. Black dott represents an outlier value of the boxplot.

The comparison of the molecular weight of the molecules detected by the two techniques is significantly different for each sampling point (all *p*-Value < 0.01, see Table S3). The weight is higher for the Coriolis sampling method with a mean at 156 g/mol than the Nalophan^®^ bag, with a mean at 103 g/mol (Figure 2).

#### 2.2.2. Behavior in Water Compartment

- Solubility in Water at 25 °C

**Figure 3:**
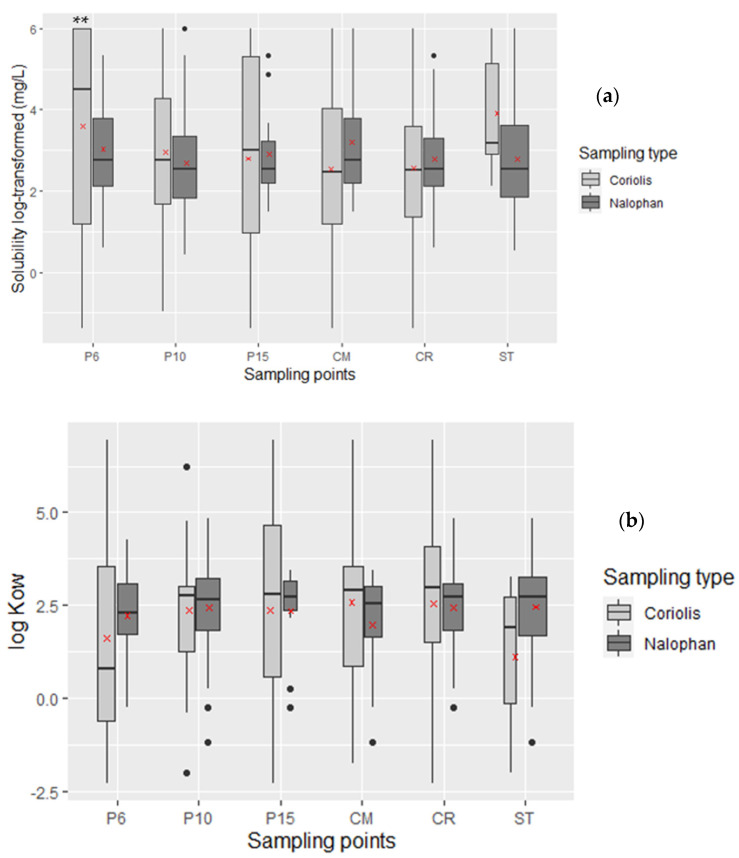
Comparison of: (**a**) solubilities in water at 25 °C according to the sampler type used. The red crosses represent the mean values. No significant difference is found between these two variables except for P6; (**b**) the octanol/water partition coefficient according to the method used. No significant difference is found between these two variables. ** mean *p* value < 0.01. Black dott represents an outlier value of the boxplot.

The solubilities of the molecules found with each technique are different. Indeed, the solubility range is greater with the Coriolis micro than with the Nalophan^®^ bag, with no significant difference except for P6, while the medians are quite similar (Figure 3a). It is interesting to note the solubilities of the molecules obtained with the Coriolis micro are not higher than those obtain with the Nalophan^®^ bags, which could have been the case since the molecules sampled with the Coriolis micro are recovered in water.

- The Octanol/Water Partition Coefficient (log K_ow_)

To better understand the capacity to these molecules to bioaccumulate in organisms, the water/octanol partition coefficient (log K_ow_) was inquired. Log K_ow_ determines if molecules are more hydrophobic or lipophilic. In other words, log K_ow_ reflects the distribution capacity of organic compounds between octanol (hydrophobic/lipophilic) and water (hydrophilic). Molecules with log K_ow_ less than 3 are considered hydrophilic compounds, whereas molecules with log K_ow_ higher than 3 are considered hydrophobic compounds.

The boxplot shows a wide range of log K_ow_ from −2.3 to 7 for Coriolis micro and from −1.2 to 4.8 for Nalophan^®^ bags, the widest being for the Coriolis micro but with no significant difference (Figure 3b). However, the medians are almost identical with 2.69 for the Coriolis micro and 2.65 for Nalophan^®^ bags. The same proportion of soluble and/or lipophile molecules was obtained with 66% for the Coriolis micro and 60% with Nalophan^®^ bags.

#### 2.2.3. Behavior in Air Compartment

- The Octanol/Air Partition Coefficient (log K_oa_)

The log K_oa_ is useful to predict the partitioning behavior of organic compounds between air and environmental matrices, such as soil, vegetation, and aerosol particles. Log K_oa_ is the ratio of a chemical’s concentration in octanol to the concentration in air equilibrium. Organic molecules with log K_oa_ comprised between 6.5 and 10 have a significant potential for long-distance air transport, whereas organic compounds with log K_oa_ < 6.5 have a high volatility and cannot be deposited on the ecosystem. Finally, organic compounds with log Koa >10 have a greater potential for adsorption by the particles surface and lipids [29,30]. Log K_oa_ also gives information about bioaccumulation. Molecules with 6 < log K_oa_ < 12 have a high bioaccumulation potential for humans [30].

**Figure 4:**
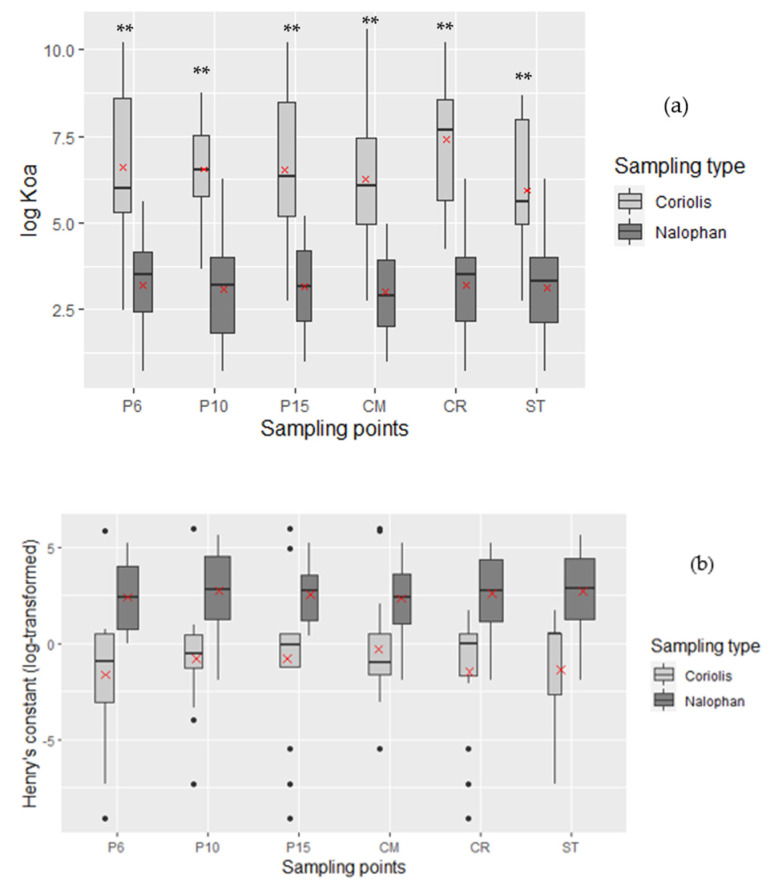

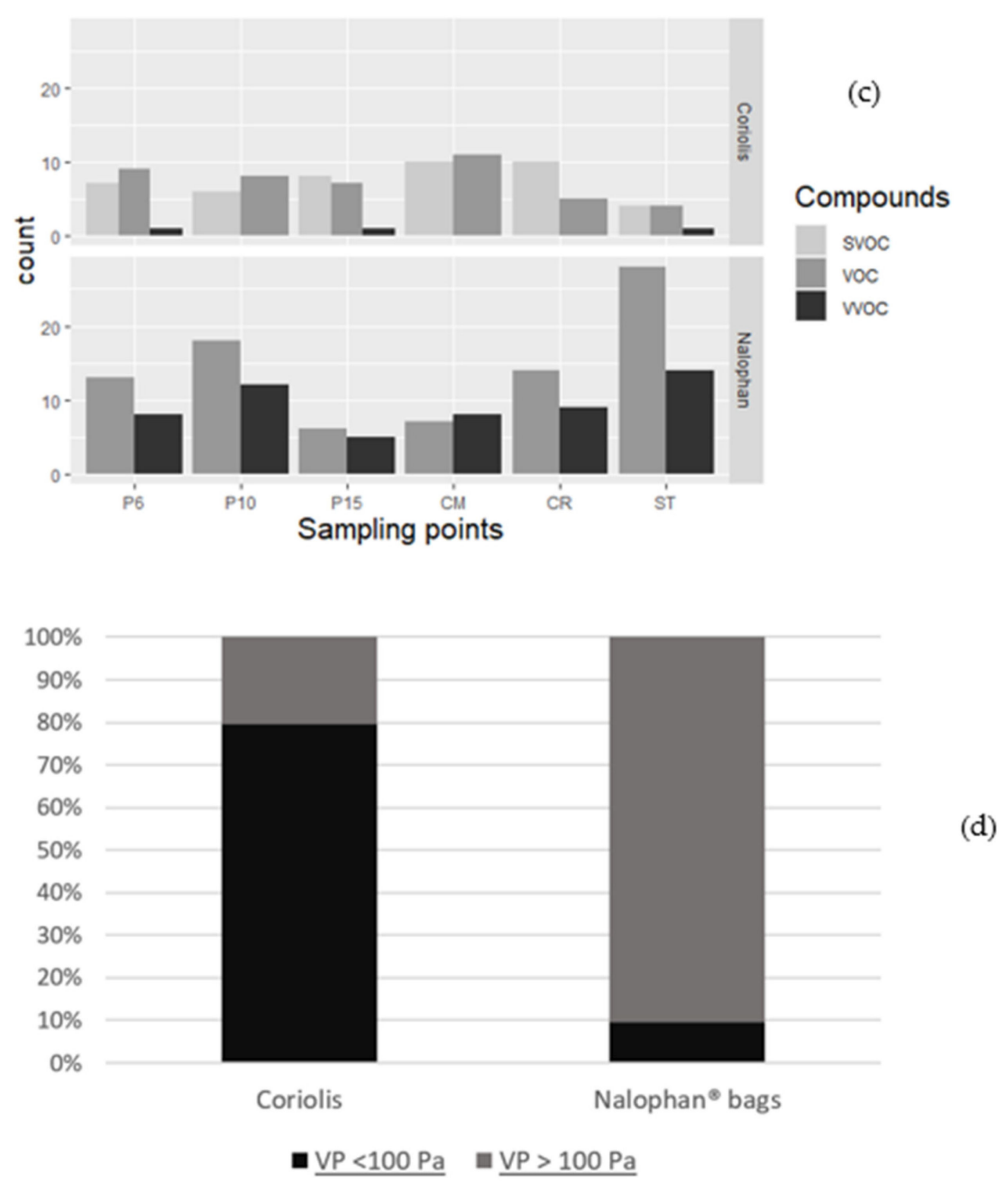
(**a**) The octanol/air partition coefficient repartition according to the method used. Log K_oa_ of molecules obtained with Coriolis micro are significantly different from those obtained with Nalophan^®^ bags (*p*-value < 0.01, Supplementary Materials: Table S3); (**b**) box plot of the log-transformed Henry’s constant in Pa.m^3^/mol molecules according to the methods used. Henry’s constants are not significantly different between those of Coriolis micro and those of Nalophan^®^ bags except for P6 (*p*-value < 0.01); (**c**) repartition of boiling points (BP) according to the method use. Figure represents BP comprised between 260 °C and 400 °C for semi volatile organic compound (SVOC); BP comprised between 100 °C and 260 °C for volatile organic compound (VOC) and BP below 100 °C for very volatile organic compound (VVOC), no significant differences were found; (**d**) vapor pressure superior and inferior to 100 Pa to determine volatility of each compound found with both techniques, no significant differences were found. Black dott represents an outlier value of the boxplot; ** mean *p* value < 0.01.

Figure 4a highlights log K_oa_ repartition for each method and shows repartitions ranging from 2.494 to 10.603 for the Coriolis micro and from 0.712 to 6.255 for Nalophan^®^ bags. The medians are different with 6.26 and 3.44 for the Coriolis micro and Nalophan^®^ bags, respectively. Values obtained for Nalophan^®^ bags are significantly different from the Coriolis values (*p*-values < 0.01). Log K_oa_ < 6.5, which defined high volatility, characterized all the molecules obtained with Nalophan^®^ bags against only 52% of molecules obtained with the Coriolis micro. Molecules sampled with the Coriolis micro had other particularities, namely potential for long distance air transport (6.5 < log K_oa_ < 10), which concern 43% of the molecules. A small proportion of molecules obtained with the Coriolis micro (4.5%) had log K_oa_ >10, which means a greater potential to be adsorbed by the particles surface and lipids.

In conclusion, molecules found with the Coriolis micro had higher log K_oa_ than molecules identified with Nalophan^®^ bags. This means that molecules found with the Coriolis had potential for long-distance air transport, bioaccumulation in humans, and adsorption to air particles. Molecules found with Nalophan^®^ bags were more volatile than molecules found with the Coriolis.

- Henry’s Constant

Henry’s constant characterizes the capacity of a substance to split between the two phases of a binary air/water system, Henry’s constants higher than 10 Pa.m^3^/mol allows to predict a volatilization of the substance from water bodies, while Henry’s constant comprised between 0.1 to 10 Pa.m^3^/mol allows to predict a slow volatilization [31]. Molecules retained by the two techniques are represented in function of their Henry’s constant distribution. Box plot highlighted that the individual’s repartition is not significantly different for each method, except for P6 (*p*-value < 0.01), even if Nalophan^®^ bags values are higher than values obtained for the Coriolis (Figure 4b). It reveals that median values were different with 2.9 and −0.53 for Nalophan^®^ bags and the Coriolis micro, respectively. The Henry’s constant repartition shows that 84% of molecules found with the Coriolis micro appears to have a Henry’s constant lower than 10 Pa.m^3^/mol (equal to 1 in log-transformed on the figure), which means slow volatilization, against 21% with Nalophan^®^ bags (data not shown). Conversely, 16% and 79% of molecules found with the Coriolis micro and Nalophan^®^ bags, respectively, had a Henry’s constant greater than 10 Pa.m^3^/mol. Consequently, molecules found with Nalophan^®^ bags are more volatile than molecules found with the Coriolis micro.

- Boiling Point (BP)

A VOC is defined with a BP ranging from 50–100 °C to 240–260 °C, according to WHO [12], and a semi volatile organic compound (SVOC) has a BP ranging from 240–260 °C to 380–400 °C. A very volatile organic compound (VVOC) has a BP lower than 50–100 °C [13].

All molecules found with Nalophan^®^ bags had a BP below 260 °C (Figure 4c). Indeed, 35 molecules found with Nalophan^®^ bags had BP comprised between 100 °C and 260 °C, which is a characteristic of VOC, while 17 molecules had a BP below 100 °C, which corresponds to VVOC. On the other hand, 21 molecules found with the Coriolis micro had a BP comprised between 260 °C and 400 °C, which is a characteristic of semi volatile molecules. However, no significant differences were obtained between values of Nalophan^®^ bags and the Coriolis micro. Moreover, the same number of molecules had a BP comprised between 100 °C and 260 °C and only 2 with a BP lower than 100 °C. In other words, the Coriolis micro enables to sample SVOC, VOC, and slightly VVOC, while Nalophan^®^ bags permit to samples mainly VOC and VVOC.

- Vapor Pressure (VP)

A vapor pressure greater than 100 Pa characterizes a volatile compound and a vapor pressure comprised between 0.001 and 100 Pa characterizes a semi volatile compound [32].

There are 79.6% of the Coriolis micro molecules with vapor pressure <100 Pa, against 10% for Nalophan^®^ bags. Inversely, 90.4% of Nalophan^®^ bags molecules had a vapor pressure >100 Pa (Figure 4d). This supports the fact that the molecules obtained with the Nalophan^®^ bags are considered as volatile, while the Coriolis micro allows to sample semi-volatile molecules.

These parameters allow to confirm that molecules found with Nalophan^®^ bags are more volatile than the ones found with the Coriolis. Molecules found with the Coriolis are defined as semi volatile compounds and can have a more complex behavior. On the other hand, molecules obtained with the Coriolis micro can be adsorbed on particles and potentially fall back into a compartment different from their origin. These molecules can remain close to their initial site or be subjected to long distance air transport.

### 2.3. Sampling in Controlled Air

To better understand the type of molecules collected by each of the two methods, we carried out samples in controlled air. These samplings allow us to avoid the variations which can be present on the field, and can be different according to the sampling points (wind, temperature, etc.)

**Figure 5:**
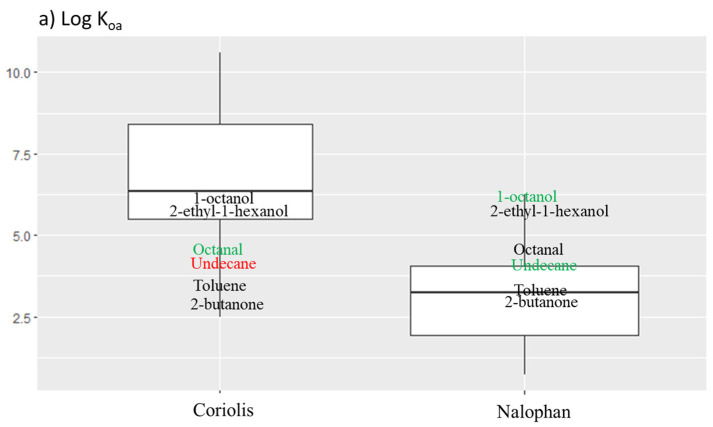

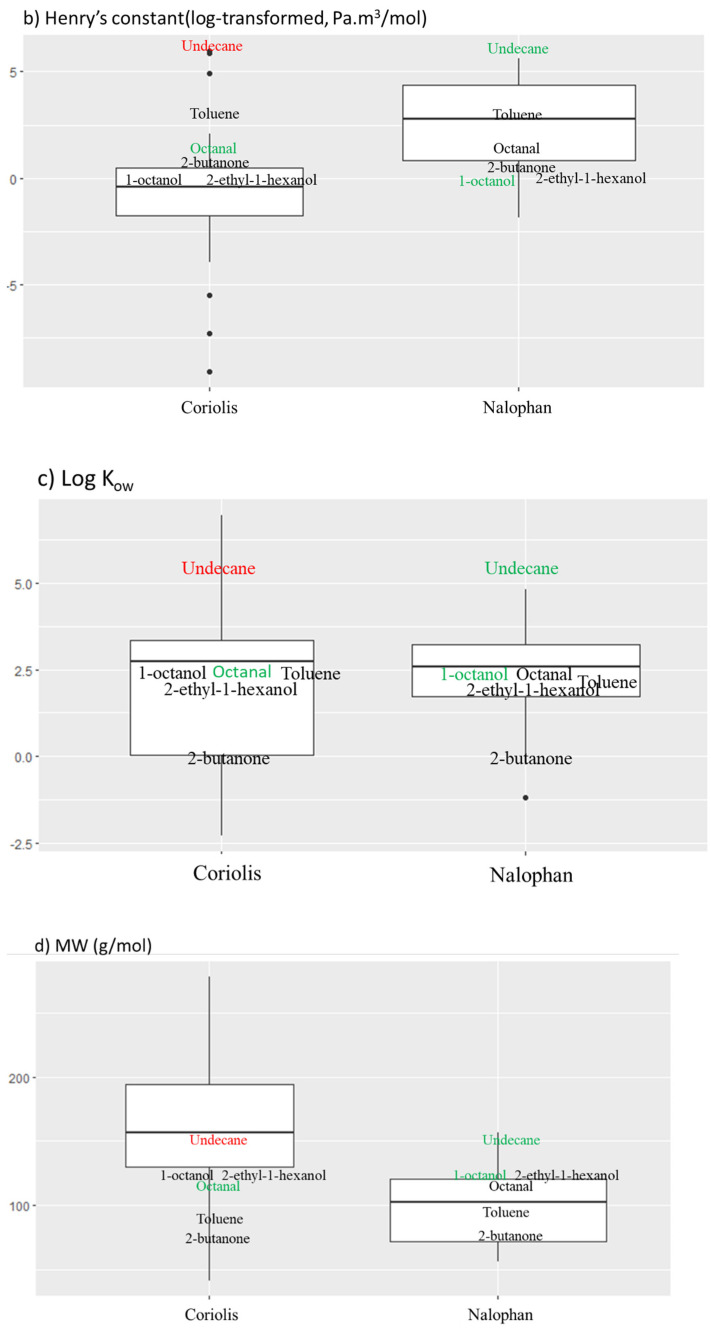

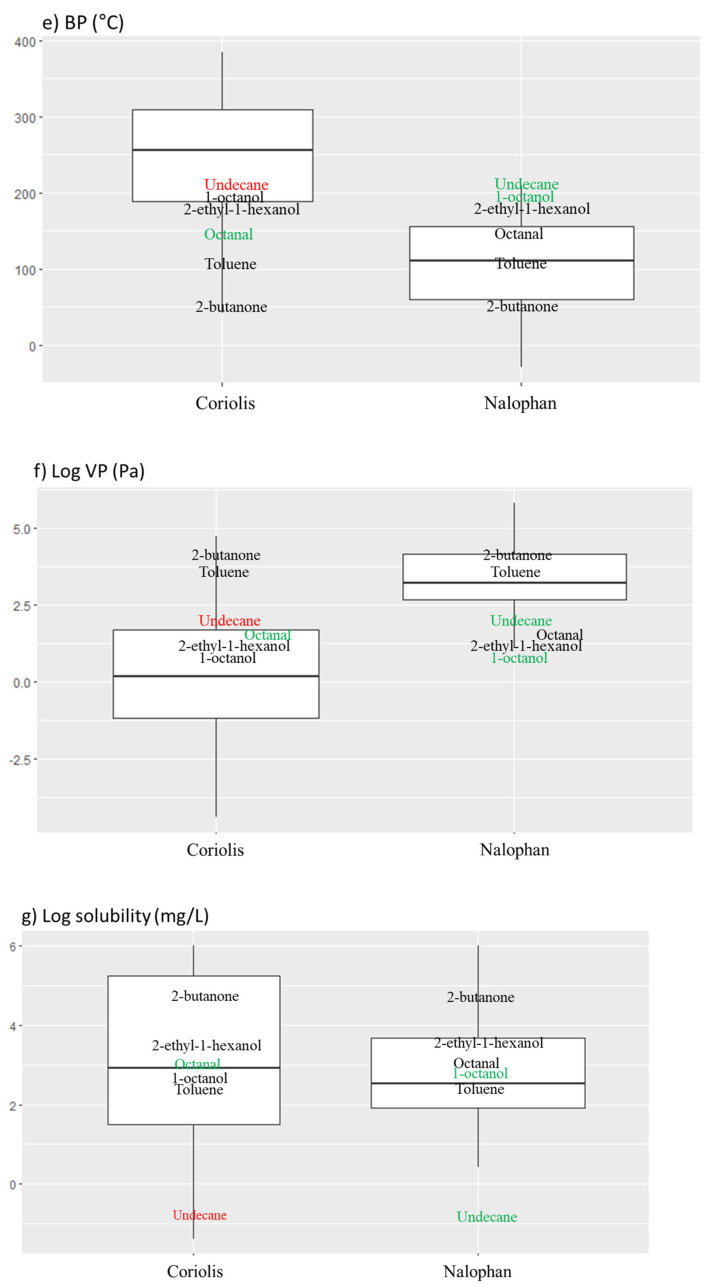
Box plot for each property previously presented: (**a**) log K_oa_, (**b**) log-transformed Henry’s constant, (**c**) log K_ow_, (**d**) molecular weight (MW), (**e**) boiling point (BP), (**f**) log vapor pressure (VP), and (**g**) log- transformed solubility (mg/L) with position of each molecule present in solution used to make artificial contaminated air. The molecules in green color are the molecules found only during the laboratory test, the molecules in red color are the molecules found only during the field. Black dott represents an outlier value of the boxplot.

A sampling test with the Coriolis micro and Nalophan^®^ bags was carried out in controlled indoor air. Seven molecules were present in a standardized solution used to make artificial contaminated air. Among these molecules, two were found in the field only with the Coriolis micro (1-octanol and undecane), two were found in the field only by Nalophan^®^ bags (toluene and 2-butanone), and two were found by both techniques (octanal and 2-ethyl-1-hexanol). The results show that the Coriolis micro sampled three molecules out of the four sampled in the field (Table 1). Among the expected molecules, only undecane was not sampled with the Coriolis micro in controlled air. Indeed, the Coriolis micro has sampled 1-octanol, octanal, and 2-ethyl-1-hexanol, in all samples (*n* = 3). Nalophan^®^ bags has sampled all the molecules present in the solution (7 out of 7 molecules). 1-octanol and undecane were not sampled by Nalophan^®^ bags in the field, while they were detected with the Coriolis micro. Sampling in controlled air showed a slight lack of sensitivity for the Coriolis micro, which did not detect a molecule present in the mixture in the three samples. Concerning Nalophan^®^ bags, all the molecules were found in controlled air but not in the field. This may imply a higher detection limit not allowing to detect some compounds presents in the environment, or a matrix effect. The selected molecules have their physico-chemical properties in the range of the detection capacities of each technique, as seen previously (Figure 5). All the properties among partage coefficient octanol/air, octanol/water, Henry’s constant, molecular weight, boiling point, vapor pressure, and solubility, seem to influence the ability of the Coriolis micro or Nalophan^®^ bags to sample a type of molecule. Indeed, all the molecules are included in the box plot, except one extreme, which is undecane for both techniques and toluene and 2-butanone for the Coriolis micro. Undecane has a low solubility in water (0.03 mg/L), which can explain why we do not find it using the Coriolis micro. The undecane was already found in the air using the Coriolis micro, but only twice in the same campaign (9 other campaigns were developed within the framework of another study and no undecane was detected: data not shown). Octanal is a widely present molecule that can be found in high concentrations in limes, mandarins, oranges, and other different foods [33,34]. Aldehyde (and octanal) are also known to be uptaken by acidic particles and form secondary organic aerosols (SOA), but this phenomenon implies a high concentration of octanal present in the atmosphere [35,36]. Moreover, it is important to note that the controlled air tests do not simulate the presence of particles in the air, while the Coriolis micro is used to sample the (bio)particles.

## 3. Discussion

A sampling method usually dedicated to the collection of microorganisms was tested. The technique is a cyclonic impaction method. According to the manufacturer’s information, particles are collected from 0.5 µm. The compounds detected by this method can be either compounds dissolved into the water or compounds bound to the particles. It was expected that the evaluated method would retain the polar and less volatile molecules, with possibly other compounds retained on the particles, while the reference method would present the more volatile molecules. However, it is not easy to provide the impact of sampling a large volume of air. The manipulations proposed in our study allow us to confirm our hypothesis.

The results of this study showed a great complementarity in the two sample methods in terms of physico-chemical properties, such as molecular weight and the octanol/air partition coefficient. More specifically, the Coriolis micro allows to sample SVOCs, VOCs, and to a lesser extent VVOCs. The molecules obtained with the Coriolis micro have shown other properties, such as long-distance potential and adsorption on particles. Additionally, Nalophan^®^ bags were able to collect VOCs and VVOCs.

Among the molecules found with both techniques, some of them are classified as of concern and require further investigation to assess their possible impact on the environment.

Among the emerging pollutants of interest, two in particular have attracted our attention, such as pyridine or methyl vinyl ketone (MVK). Pyridine is ubiquitous and is used as a solvent and intermediate in the production of agricultural chemicals, drugs, and paints [37]. Pyridine is also used in other processes i.e., a derivatization agent associated with methyl imidazole for converting non-volatile substances in volatiles targets [38]. Exposure to pyridine and its derivatives, such as 3-picoline, 2-aminopyridine, 4-aminopyridine, etc., has harmful effects on the liver, kidneys, reproductive functions, and the immune system. Furthermore, it has potential carcinogenicity and has toxicity to aquatic life [39]. Pyridine has a very high half-life when released in the atmosphere [40] and it is also included in the list of chemicals subjected to EPCRA in the section, toxic chemicals [41]. MVK is one of the two major compounds produced during the oxidation of isoprene by the OH and is ozone-initiated, which is the largest atmospheric emission flux among all non-methane VOCs [42]; it may also have anthropogenic sources [43]. MVK can be hazardous to human health with upper respiratory tract effects [44] and is on the consolidated list of chemicals subject to the emergency planning and community right-to-know act (EPCRA), in the section, extremely hazardous substances, from USEPA [44].

Currently, atmospheric exposure risk assessments are performed on a limited number of methods, most often using targeted analyses [45,46,47,48,49]. Non targeted analyses are beginning to be used, particularly for the food matrices [50,51], and have shown their usefulness in facilitating provisional safety assessments and in finding new emerging chemicals [28,52]. Besides the non-targeted analysis, we show here that the development of alternative sampling methods is also essential for a more comprehensive risk assessment. The use of the Coriolis has allowed to highlight other molecules of interest and those emerging, such as phthalates (benzyl butyl phthalate, diisobutyl phthalate, dimethyl phthalate, and diethyl phthalate), phenol, pyridine, triethyl phosphate, and 1,2-benzothiazole, among other.

## 4. Materials and Methods

### 4.1. Sampling Points

**Figure 6:**
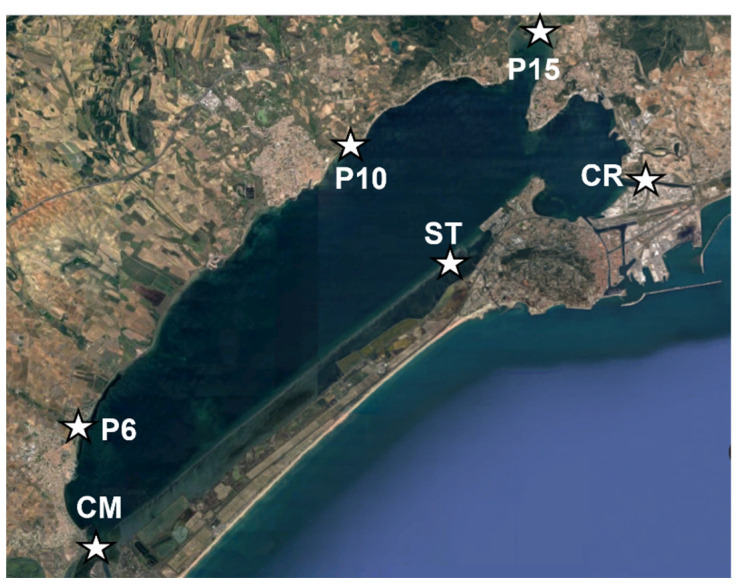
Distribution of sampling points around Thau lagoon.

The sampling was carried out in the south of France around the Thau lagoon. As shown in Figure 6, six different sampling points were selected around the lagoon, according to other studies previously developed in the framework of regulatory monitoring [53,54]. Among several sampling sites (from P1 to P20), three points which are located on the main rivers entering the lagoon were selected. P6 corresponded to the end of the Fontanilles stream, P10 corresponded to the end of the Pallas stream, and P15 corresponded to the end of the Vène stream. To continue in the follow-up of the major tributaries arriving in the lagoon, we decided to add the point CM, corresponding to the end of the canal of midi, and the point CR, corresponding to the beginning of the canal of Rhône. Finally, a last sampling point, directly in the lagoon and near the tourist town of Sète, was chosen and called ST.

### 4.2. Samples Collection and Analyses

At each point, air samples were collected using two different techniques. The first one consists of collecting air using laboratory-made 40 L Nalophan^®^ bags and a lung chamber, according to the NF X 43-104 air sampling standard. The second technique involves using the Coriolis Micro (Bertin instruments, France) to collect air particles in 15 mL of distilled water for 40 min at 300 L/min. These sampling conditions correspond to the usual conditions of use in the field. The result is a difference in the sampling volume. Then, extractions and analyses were performed on each type of sample (Table 2). One sample was sampled at each site by each technique in a duplicate experiment.

The generation of the controlled atmosphere is conducted as follows: volatilization of liquid solution of 6 compounds (Table 3) in a 20 L/min air flow; then dilution of this air flow in a 417 L/min air flow to reach the required concentration (417 L/min is the maximum air flow that can be set in the dilution pilot). We use a high flow rate because of the Coriolis sampling constraints. Three samples were realized by each technique in duplicate experiment.

Sampling using the Coriolis micro and Nalophan^®^ bags were performed as described previously. Each sampling was performed in triplicate. The volumes of liquid that were withdrawn with the Coriolis micro were 15, 22, and 30 mL, respectively.

### 4.3. Estimated Data

The physico-chemical properties of each compound were determined using the Estimation Programs Interface Suite 4.11 (EPISuiteTM) [56].

KOWINTM was used for estimating log K_ow_ with an atom/fragment contribution method, HENRYWINTM was utilized to determine Henry’s constant using the group and bound contribution. The vapor pressure and boiling points for each compound has been determined by the MPBPVPTM tool and KOAWINTM was used to estimate the K_oa_, using the ration of the log K_ow_ from KOWINTM and the dimensionless Henry’s Law constant from HENRYWINTM.

To obtain all these values, we entered the Simplified Molecular-Input Line-Entry System (SMILES) of each molecule in the software.

### 4.4. Statistical Analysis

A statistical analysis and drawing were performed using the R software, version 4.1.1 by R core team [57]. The data were analyzed by the Shapiro-Wilk test to determine if our data followed a normal distribution. If data followed a normal distribution a *t*-test of Student for unpaired data was applied, if not a Test U from Mann-Whitney was chosen. For all statistical tests a P value threshold of 0.05 was used. A Venn diagram was realized using the software interractivenn.net (date of access: 01/06/2022) [58].

## Figures and Tables

**Table 1:** Molecules detected (yes) or not (no) according to sampling type and place (lab/field), (*n* = 3).

Molecules	Field		Laboratory	
	Coriolis Micro	Nalophan^®^ Bags	Coriolis Micro	Nalophan^®^ Bags
1-octanol	yes	no	yes	yes
2-butanone	no	yes	no	yes
2-ethyl-1-hexanol	yes	yes	yes	yes
Octanal	no	yes	yes	yes
Toluene	no	yes	no	yes
Undecane	yes	no	no	yes

**Table 2:** Methods collection and analysis used for air samples.

Stages	Description	Nalophan^®^ Bag	Coriolis Micro
**Sampling**	Instruments	Laboratory-made 40 L Nalophan^®^ bag + lung chamber (NF X 43-104)	Coriolis micro (Bertin instruments, France)
	Quantity of air sampled	40 L	12,000 L
**Extraction**	Recovery unit	18 L are pumped through AirToxic (Perkin Elmer, Waltham, MA, USA) multi-bed sorbent tubes	15 mL of distilled water in an amber glass vial. SBSE ^a^ with 10 mm Twister (Gerstel, Germany) magnetic stir bars coated with polydimethylsiloxane (PDMS) (0.5 mm film thickness) or ethylene glycol Silicone (EGS) *. The method was an adaptation of the Berrou et al., 2020 study [55]
	Flow rate	100 mL/min	-
	Agitation-duration	-	600 rpm during 2 h
**Desorption**	Instruments	Turbomatrix, PerkinElmer, USA	Thermal desorption unit (TDU) and cooled injection system (CIS) (Gerstel, Germany)
	Steps-Temperature-duration	250 °C for 15 min	TDU: 220 °C for 5 min CIS: −10 °C for 1 min ramped to 270 °C at a heating rate of 12 °C per second and held for 2.5 min in spitless mode
**Analysis**	Instruments	Clarus 680, Perkin Elmer, USA	Agilent 7890B gas chromatograph
	Columns	Elite-5-ms (60 m × 0.25 mm × 1 µm, Perkin Elmer, USA)	ZB-5MSplus fused silica capillary column (30 m × 0.25 mm × 0.25 µm, Phenomenex, Torrance, CA, USA)
	Gas-flow rate	Helium-constant pressure of 30 psi	Helium-0.8 mL/min
	Oven program	9 min at 40 °C, a ramp at 15 °C/min until 90 °C hold 4 min, then a ramp at 10 °C/min until 250 °C hold 15 min	4 min at 40 °C, a ramp at 6 °C/min until 300 °C, hold 1 min
**Detection**	Instruments	SQ8T model mass spectrometer (Perkin Elmer, USA)	5977A mass spectrometer (Agilent, Santa Clara, CA, USA)
	Mode	Electronic impact at 70 eV	Electronic impact at 70 eV
	Acquired mass	20 to 350 amu	33 to 350 amu
	Analysis type	Scan mode	Scan mode
	Identification	NIST library	NIST library

^a^ SBSE = Stir Bar Sorptive Extraction, amu = atomic mass unit. * Stir bars were conditioned prior to use according to the manufacturer’s instructions.

**Table 3:** Information about substances contained in the solution used for air-controlled sampling.

CAS	Substances	Family	Molecular Weight (g/mol)	Concentration (mg/m^3^)	References ^1^
78-93-3	2-butanone	Ketone	72.11	3.59	VWR (25643.294)
108-88-3	Toluene	Aromatic	92.14	1.95	Supelco (1.00849.2500)
124-13-0	Octanal	Aldehyde	128.21	2.76	Acros (199481000)
104-76-7	2-ethyl-1-hexanol	Alcohol	130.23	2.81	Acros (118530010)
111-87-5	1-octanol	Alcohol	130.23	3.69	Fluka (74852)
1120-21-4	Undecane	Alkane	156.31	2.48	Acros (140665000)

^1^ All the standards have a purity higher than 99% (chromatography grade).

## Supplementary Materials

The following supporting information can be downloaded at: https://www.mdpi.com/article/10.3390/molecules27196462/s1, Table S1: physico-chemicals properties of molecules found with the Coriolis micro; Table S2: physico-chemicals properties of molecules found with Nalophan^®^ bags. Table S3: details of statistical tests.

## Sample Availability

Samples of the compounds are available from the authors.
