# Supplementary material for: Evaluation of Coriolis Micro Air Sampling to Detect Volatile and Semi-Volatile Organic Compounds
Source: Molecules. 2022 Sep 30;27(19):6462. doi: 10.3390/molecules27196462 (PMC9572053; doi:10.3390/molecules27196462)
Supplement: Supplementary file 1 [file molecules-27-06462-s001.zip › molecules-1874613-supplementary.pdf]

Table S1. Physico-chemicals properties of molecules found with Coriolis micro.

Coriolis Molecules	MW (g/mol)	Log K_{ow}	Log K_{oa}	Boiling Point (°C)	Solubility (mg/L)	Vapor Pressure (Pa)	Henry's Constant (Pa.m³/mol)
Nonanal	142.24	3.27	4.966	191	131.6	49	50
1,2-ethanediol monoformate	90.08	−1.148	5.17	144.76	1×10^{6}	241	1.18×10^{-3}
1,2-ethanediol monoacetate	104.11	−0.601	5.86	188	1×10^{6}	20.1	8.62×10^{-4}
Methylal	76.1	−0.195	2.494	42	1.33×10^{5}	$5.31 x 10^{4}$	5.13
2-ethyl-1hexanol	130.23	2.732	5.627	184.6	1379	18.1	3.14
1-octanol	130.23	3	5.707	195.1	814	10.6	3.14
Diethyl carbitol	162.23	0.5	5.324	188	3.11×10^{4}	69.5	3.72×10^{-2}
(S)-(+)-6-methyl-1-octanol	144.26	3.223	5.994	208.49	459.7	5.88	4.17
Undecane	156.31	5.744	3.281	195.9	0.2571	54.9	7.13×10^{5}
Tetraethylene glycol	194.23	−2.023	8.68	328	1×10^{6}	0.0062	4.97×10^{-8}
Pyridine	79.1	0.804	4.34	115.2	7.3×10^{5}	2.77×10^{3}	0.714
Triethylene glycol	150.18	−1.748	8	285	1×10^{6}	0.176	3.2×10^{-6}
1,3-dioxolane-2-methanol	104.11	−0.405	6.96	178.11	8.15×10^{5}	36.1	1.1×10^{-4}
Dodecanoic acid	200.32	4.998	8.42	298.9	12.76	0.00213	0.944
Pentaethylene glycol	238.28	−2.297	10.207	353.51	1×10^{6}	3.98×10^{-5}	7.73×10^{-10}
1,2-benzenedicarboxylic acid, bis(2-methylpropyl) ester	278.35	4.463	8.762	296	5.061	0.322	0.124
7,9-Di-tert-butyl-1-oxaspiro(4,5)deca-6,9-diene-2,8-dione	276.38	3.554	8.614	384.56	15.5	2.06×10^{-4}	0.0213
n-Hexadecanoic acid	256.43	6.962	9.888	351.5	0.04	5.07×10^{-5}	2.93
Phenol	94.11	1.513	6.149	181.8	2.62×10^{4}	46.7	5.68×10^{-2}
Benzaldehyde, 2,5-dimethyl-	134.18	2.805	5.974	220	356.1	17.5	1.66
1-dodecanol	186.34	4.77	7.175	259	6.898	0.113	9.76
Oxime, methoxy-phenyl	151.17	1.162	7.927	276.2	7627	0.0779	4.24×10^{-4}
Dodecane	170.34	6.235	3.648	216.3	0.1099	18	9.47×10^{5}
Benzoic acid, 4-ethoxy, ethyl ester	194.23	2.891	6.72	275	39.44	0.653	0.367
Cyclopentaneacetic acid, 3-oxo-2-pentyl, methyl ester	226.32	2.975	7.668	309.32	91.72	0.158	5.08×10^{-2}
Benzenesulfonamide, N-butyl	213.3	2.309	6.362	314	398	0.0189	0.22
Acetonitrile	41.05	−0.146	2.753	81.6	1.37×10^{5}	1.18×10^{4}	3.1
Triethyl phosphate	182.16	0.872	5.493	215.5	1.12×10^{4}	52.4	5.91×10^{-2}
1-octene, 6-methyl	126.24	4.551	3.015	127.33	3.583	1.83×10^{3}	8.5×10^{4}
1,2-Benzothiazole	135.18	2.169	5.937	220	1232	12.4	0.424
Tetradecanoic acid	228.38	5.98	9.154	326.2	0.4668	1.87×10^{-4}	1.66
Iridomyrmecin	168.24	1.892	3.884	276.6	1518	0.416	25.1
Benzamide, N,N-diethyl-4-methyl	191.28	2.258	8.33	308.11	449.4	0.0442	2.1×10^{-3}
Propanoic acid, 2-methyl, 2,2-dimethyl-1-(2-hydroxy-1-methylethyl)propyl ester	216.32	2.997	8.474	263.84	321.5	0.173	8.32×10^{-3}

Propanoic acid, 2-methyl, 1-(1,1-dimethylethyl)-2-methyl-1,3-propanediyl ester	286.42	4.911	8.324	271.5	0.9422	1.13	0.955
4-Heptanone, 2-methyl	128.22	2.147	4.229	155	1371	548	20.7
Pentadecanoic acid	242.41	6.471	9.52	339.1	0.1925	5.8×10^{-5}	2.21
Acetic acid	60.05	0.087	4.74	117.9	4.76×10^{5}	2.09×10^{3}	5.55×10^{-2}
1,2-ethanediol diformate	118.09	−0.69	3.681	174	3.96×10^{5}	172	0.105
Dodecanal	184.32	4.745	6.078	252.62	4.649	2.04	1.17×10^{2}
Dimethyl phthalate	194.19	1.663	6.698	283.7	2014	0.411	2.27×10^{-2}
Diethyl Phthalate	222.24	2.646	7.443	295	287.2	0.28	3.99×10^{-2}
2-propanol, 1-chloro-phosphate	327.57	2.886	8.503	365.49	51.85	0.00753	6.04×10^{-3}
Benzyl butyl phthalate	312.37	4.845	10.603	370	0.9489	0.0011	4.28×10^{-3}

Table S2. Physico-chemicals properties of molecules found with Nalophan® bags.

Nalophan® Bags Molecules	Log K_{ow}	Log K_{oa}	Boiling Point (°C)	Solubility (mg/L)	Vapor pressure (Pa)	Henry's constant (Pa.m³/mol)
Dichlorodifluoromethane	1.816	0.751	−29.8	258	6.45×10^{5}	2.91×10^{4}
2-butene	2.091	1.098	3.73	423.5	2.31×10^{5}	2.43×10^{4}
Butane	2.306	0.712	−0.5	135.6	2.43×10^{5}	9.82×10^{4}
2-methylbutane	2.723	0.998	27.8	184.6	9.19×10^{4}	1.3×10^{5}
Trichloromonofluoromethane	2.129	1.812	23.7	593.2	1.07×10^{5}	5.15×10^{3}
2-methyl-1-propene	2.226	1.238	−6.9	399.2	3.08×10^{5}	2.43×10^{4}
Acetone	−0.235	2.453	56	2.2×10^{5}	3.09×10^{4}	5.02
Pentane	2.797	1.078	36	49.76	6.85×10^{4}	1.3×10^{5}
Isoprene	2.58	1.882	34	338.6	7.33×10^{4}	1.24×10^{4}
3-methylpentane	3.214	1.365	63.2	31.09	2.53×10^{4}	1.75×10^{5}
2-methylpentane	3.214	1.365	60.2	66.35	2.81×10^{4}	1.73×10^{5}
Methyl tert-butyl ether	1.429	2.513	55.2	1.98×10^{4}	3.33×10^{4}	2.04×10^{2}
3-buten-2-one	0.408	3.382	81.4	6.06×10^{4}	1.22×10^{4}	2.65
Butanone	0.256	2.83	79.5	7.61×10^{4}	1.21×10^{4}	6.67
Ethyl tert butyl ether	1.92	2.88	73.1	2640	1.65×10^{4}	2.71×10^{2}
2-methyl-1-propanol	0.767	4.159	107.8	9.71×10^{4}	1.4×10^{3}	1.01
Vinyl methacrylate	1.63	3.035	115.98	4341	2.49×10^{3}	97.6
Allyl methacrylate	2.121	4.23	138.95	1470	769	19.3
2-butenal (Z)	0.601	3.239	102.2	4.18×10^{4}	4×10^{3}	5.68
1,2-dichloro ethane	1.832	2.136	83.5	6414	1×10^{4}	1.23×10^{3}
Butan-1-ol	0.841	4.229	117.7	7.67×10^{4}	8.93×10^{2}	1.01
Cyclohexane	3.176	2.162	80.7	43.02	1.29×10^{4}	2.59×10^{4}
3-ethyl-2-pentanone	1.656	3.861	127.98	4057	1.78×10^{3}	15.6
2,2-dimethyl hexane	4.159	2.07	106.8	8.576	4.53×10^{3}	3.05×10^{5}
Pentanal	1.307	3.497	103	9718	3.47×10^{3}	16.1
3,3,4-trimethyl hexane	4.577	2.37	140.5	3.349	1.18×10^{3}	4.05×10^{5}
Ethanediol	−1.2	4.071	197.3	1×10^{6}	12.3	1.32×10^{-2}
3-methylheptane	4.197	2.11	118	7.965	2.61×10^{3}	3.05×10^{5}
Toluene	2.54	3.154	110.6	573.1	3.79×10^{3}	6.02×10^{2}
2,4-dimethyl-3-pentanone	1.582	3.781	125.4	2716	1.79×10^{3}	15.6

1,3-dimethyl cyclohexane	4.011	2.75	122.5	11.67	2.85×10^{3}	4.56×10^{4}
2,3,5-trimethyl hexane	4.541	2.33	131.4	3.593	1.56×10^{3}	4.05×10^{5}
3,4-dimethyl-1-pentanol	2.167	5.19	154.62	4723	139	2.37
4-methyloctane	4.688	2.5	142.4	2.691	911	4.05×10^{5}
Ethylbenzene	3.031	3.521	136.1	228.6	1.28×10^{3}	800
o-xylene	3.088	3.662	144.5	224.1	881	665
Heptanal	2.29	4.231	152.8	1167	469	28.3
Styrene	2.895	3.838	145	343.7	853	280
m-xylene	3.088	3.662	139.1	207.2	1110	665
1-butoxy-2-propanol	0.984	6.255	171.5	4.21×10^{4}	53	1.32×10^{-2}
Alpha-pinene +	4.269	3.629	155.9	4.071	633	1.08×10^{4}
Propyl benzene	3.523	3.887	159.2	70.73	456	1.06×10^{3}
1-ethyl-2-methylbenzene	3.579	4.028	165.2	96.88	348	883
1-ethyl-4-methylbenzene	3.579	4.028	162	79.59	400	883
Benzaldehyde	1.71	4.971	179	6100	169	1.36
Beta pinene	4.347	3.532	166	7.061	391	1.63×10^{4}
Octanal	2.781	4.599	171	394.3	157	37.6
Cumene	3.449	3.817	152.4	75.03	600	1.06×10^{3}
2-ethyl-1hexanol	2.732	5.627	184.6	1379	18.1	3.14
D-limonene	4.827	3.639	176	4.581	207	3.85×10^{4}
Nonanal	3.27	4.966	191	131.6	49.3	50
Decanal	3.763	5.33	208.5	43.52	13.7	66.3

Table S3. details of statistical tests.

Properties	Sampling points	Sampling types	Shapiro-Wilk	Student	Test U
Molecular weight	P6	Coriolis micro	0.6361	0.0044	-
		Nalophan®	0.2793		
	P10	Coriolis micro	0.9036	0.0014	-
		Nalophan®	0.06		
	P15	Coriolis micro	0.6958	0.0003621	-
		Nalophan®	0.6162		
	CR	Coriolis micro	0.1965	2.33×10^{-6}	-
		Nalophan®	0.1918		
	CM	Coriolis micro	0.8877	0.00018	-
		Nalophan®	0.7911		
	ST	Coriolis micro	0.06	-	0.0008
		Nalophan®	0.018		
Solubility	P6	Coriolis micro	0.01049	-	6.8×10^{-7}
		Nalophan®	0.49		
	P10	Coriolis micro	0.6579	0.6713	-
		Nalophan®	0.2534		
	P15	Coriolis micro	0.1895	0.8903	-
		Nalophan®	0.0498		
	CR	Coriolis micro	0.4567	0.7337	-
		Nalophan®	0.2543		
	CM	Coriolis micro	0.3765	0.2745	-
		Nalophan®	0.2248		
	ST	Coriolis micro	0.1757	0.05682	-

		Nalophan®	0.05899		
Log K_{ow}	P6	Coriolis micro	0.311	0.4443	
		Nalophan®	0.4794		
	P10	Coriolis micro	0.8882	0.9099	-
		Nalophan®	0.3963		
	P15	Coriolis micro	0.4764	0.9773	-
		Nalophan®	0.004287		
	CR	Coriolis micro	0.1679	0.8853	-
		Nalophan®	0.2991		
	CM	Coriolis micro	0.6537	0.3406	-
		Nalophan®	0.03555		
	ST	Coriolis micro	0.1794	0.08845	-
		Nalophan®	0.3273		
Log K_{oa}	P6	Coriolis micro	0.6765	1.04×10^{-5}	-
		Nalophan®	0.2171		
	P10	Coriolis micro	0.8656	1.31×10^{-7}	-
		Nalophan®	0.4143		
	P15	Coriolis micro	0.6501	0.0001138	
		Nalophan®	0.7571		
	CR	Coriolis micro	0.5411	1.242×10^{-7}	
		Nalophan®	0.5744		
	CM	Coriolis micro	0.8884	2.228×10^{-6}	
		Nalophan®	0.4885		
	ST	Coriolis micro	0.4816	0.002917	
		Nalophan®	0.4552		
Henry	P6	Coriolis micro	4.1×10^{-7}	-	0.0004294
		Nalophan®	1.5×10^{-7}		
	P10	Coriolis micro	3.037×10^{-5}	-	0.2753
		Nalophan®	8.27×10^{-9}		
	P15	Coriolis micro	3.05×10^{-6}	-	0.1993
		Nalophan®	3.226×10^{-5}		
	CR	Coriolis micro	5.89×10^{-6}	-	0.04349
		Nalophan®	3.92×10^{-8}		
	CM	Coriolis micro	1.329×10^{-6}	-	0.3785
		Nalophan®	1.885×10^{-6}		
	ST	Coriolis micro	0.001184	-	0.1993
		Nalophan®	5.789×10^{-11}		
Boiling point	P6	Coriolis micro	0.4633	0.7825	-
		Nalophan®	0.747		
	P10	Coriolis micro	0.4633	0.431	-
		Nalophan®	0.6369		
	P15	Coriolis micro	0.253	0.593	-
		Nalophan®	0.2983		
	CR	Coriolis micro	1	0.6258	-
		Nalophan®	0.6878		
	CM	Coriolis micro	0.1572	0.6698	-
		Nalophan®	0.2196		
	ST	Coriolis micro	2.2×10^{-6}	-	0.306
		Nalophan®	1		
Vapor pressure	-	Coriolis micro	0.4633	1	-

Nalophan®	0.1939
